# New Insights into the Nrf-2/HO-1 Signaling Axis and Its Application in Pediatric Respiratory Diseases

**DOI:** 10.1155/2019/3214196

**Published:** 2019-11-19

**Authors:** Xueyan Zhang, Ming Ding, Ping Zhu, Huanlei Huang, Quan Zhuang, Jie Shen, Yufeng Cai, Mingyi Zhao, Qingnan He

**Affiliations:** ^1^Department of Pediatrics, The Third Xiangya Hospital, Central South University, Changsha, Hunan Province 410013, China; ^2^Xiangya School of Medicine, Central South University, Changsha, Hunan Province 410013, China; ^3^Xiangya School of Public Health, Central South University, Changsha, Hunan Province 410013, China; ^4^Guangdong Provincial People's Hospital, Guangdong Academy of Medical Sciences, Guangdong Cardiovascular Institute, Guangzhou, Guangdong 510100, China; ^5^Transplantation Center of the 3rd Xiangya Hospital, Central South University, Changsha, Hunan 410013, China; ^6^Xiangya School of Life Science, Central South University, Changsha, Hunan Province 410013, China

## Abstract

Respiratory diseases are one of the most common pediatric diseases in clinical practice. Their pathogenesis, diagnosis, and treatment are thus worthy of further investigation. The nuclear factor erythroid 2-related factor 2/heme oxygenase 1 (Nrf2/HO-1) signaling axis is a multiple organ protection chain that protects against oxidative stress injury. This signaling axis regulates anti-inflammation and antioxidation by regulating calcium ions, mitochondrial oxidative stress, autophagy, ferroptosis, pyroptosis, apoptosis, alkaliptosis, and clockophagy. This review presents an overview of the role of the Nrf2/HO-1 signaling axis in the pathogenesis of pediatric respiratory diseases and the latest research progress on this subject. Overall, the Nrf2/HO-1 signaling axis has an important clinical value in pediatric respiratory diseases, and its protective effect needs further exploration.

## 1. Introduction

Nearly 6 million children under the age of 5 die each year globally. In 2016, the three leading global causes of death in children of this age group were lower respiratory infections, neonatal preterm birth complications, and neonatal encephalopathy due to birth asphyxia and trauma, combined resulting in 1.80 million deaths [1]. Pediatric respiratory infection is more likely to cause airway obstruction or respiratory failure and other serious consequences than adult infections because children may have incomplete airway development, a narrow airway with few alveoli, and poor respiratory regulation function. In addition, many studies have linked air pollutants to respiratory diseases and symptoms in children. In recent years, worsening air quality has increased the prevalence of respiratory diseases, and children are more vulnerable to air pollutants because they spend more time outdoors. For example, Mazenq et al. found that short-term exposure to particulate matter of less than 10 micrometers (PM10) measured near the homes of children was linked with an increase in asthma emergency room visits [2].

For the advancement of clinical therapy, new treatment ideas and preventive measures need to be explored and applied. Research has revealed that the nuclear factor erythroid 2-related factor 2/heme oxygenase 1 (Nrf2/HO-1) signaling axis is involved in pediatric respiratory diseases. This review summarizes the research on the Nrf2/HO-1 signaling axis and our practical work experience on children's respiratory diseases. Peptides with the specific tumor necrosis factor receptor I (TNFRI) sequence such as TNFRI206-211 bind to TNF and inhibit TNF-induced p38 activation, respiratory burst, cytokine production, and adhesion receptor expression. TNFRI206-211 can also inhibit respiratory syncytial virus-induced lung inflammation in mice [3]. Yuan et al. found that NOX4/ROS and CSE/H(2)S signaling pathways may play an important role in the pathogenesis of chronic obstructive pulmonary disease- (COPD-) related pulmonary hypertension (PH) by detecting the expression of NOX4 and CSE proteins in lung tissue by immunohistochemistry and measuring the thickness of pulmonary arteriole walls [4].

Nrf2 is a member of the leucine zipper transcriptional activating factor cap-n-collar (CNC) family and consists of seven domains, Neh1-7; its negative regulator, Kelch ECH-associating protein 1 (Keap1), contains five domains, namely, NTR, BTB, IVR, DGR, and CT [5]. In the physiological state, the Neh2 domain of Nrf2 binds to the DGR region of Keap1 and is present in the cytoplasm. With the participation of Keap1 functional domains BTB and IVR, the Nrf2 is degraded by Cul3/Rbx1 E3 ubiquitin to maintain a stable concentration [6]. Nrf2 forms a heterodimer through the Neh1 domain with the Maf protein and binds to the antioxidant response element (ARE) to regulate the activities of target genes such as *SOD*, *CAT*, and those of phase II detoxifying enzymes to remove harmful molecules such as reactive oxygen species (ROS) [7].

**Figure 1:**
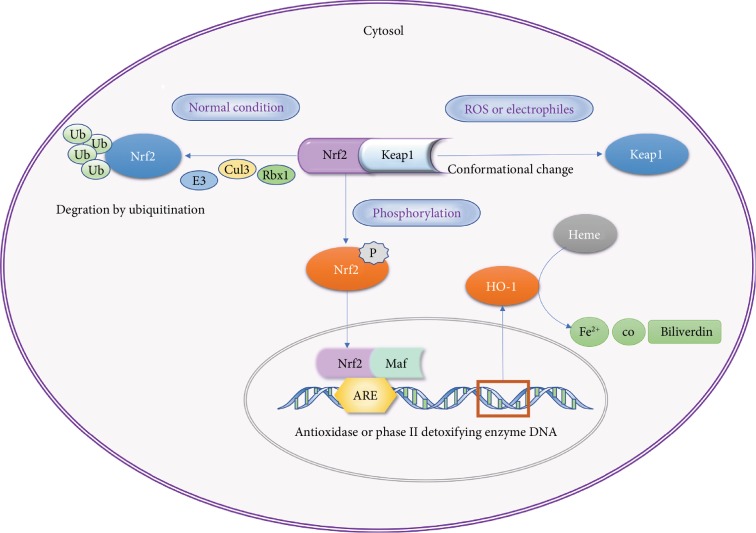
Nrf2/HO-1 pathway.

HO-1 is one of the detoxifying enzymes in phase II and the downstream signaling axis induced by it has a protective effect against oxidant stress in multiple organs. HO-1 combined with NADPH and cytochrome P450, through biliverdin reductase- (BVR-) mediated degradation of heme and the formation of biliverdin, CO, and Fe^2+^, constitute endogenous protective substances that regulate numerous cellular activities such as cell oxidation and apoptosis [8]. The complete Nrf2/HO-1 signaling axis is involved in many functions such as calcium regulation, mitochondrial oxidative stress, iron death, scorching death, autophagy, programmed cell necrosis, alkali death, and biological clock regulation. The possible pathway of the Nrf2/HO-1 signaling axis is depicted in Figure 1.

Nrf2 activity is normally suppressed in the cytosol by specific binding to the Keap1 chaperone [9]. However, upon stimulation by electrophilic agents or compounds that possess the ability to modify thiol groups [10], the Keap1-mediated repression of Nrf2 activity is lost, allowing the translocation of Nrf2 to the nucleus and the potentiation of the ARE response [9]. This mechanism of gene activation leads to the synthesis of highly specialized proteins that efficiently protect mammalian cells from various forms of stress and consequently reduce the propensity of tissues and organisms to develop disease or malignancy [11]. Proteins induced by Nrf2 include *γ*-glutamyl cysteine synthetase [12, 13], glutathione S-transferases [14], NADP(H):quinone oxidoreductase [15], and HO-1 [16], which generate the antioxidant biliverdin and the signaling molecule CO.

The Nrf2 pathway plays both “Ying and Yang” roles on the outcome of diseases. For example, although Nrf2 may decrease cancer progression, it may also be involved in various carcinogenic signal axes, which will all be described later in this review. In addition, irradiation inhibits osteoblast differentiation and mineralization of MC3T3-E1 cells through the oxidative stress-mediated activation of the Nrf2/HO-1 pathway [17].

## 2. Mechanism of the Nrf2/HO-1 Signaling Axis

### 2.1. Calcium Regulation

Cholesterol stimulation upregulates the expression of HO-1 in a time-dependent manner via the activation and translocation of Nrf2, activation of the mitogen-activated protein kinase (MAPK)/extracellular signal-regulated kinase (ERK) signaling pathway, and an increase in intracellular Ca^2+^ concentrations ([Ca^2+^]i) [18]. An excessive increase in the intracellular Ca^2+^ concentration can lead to oxidative stress and body dysfunction through various mechanisms such as intracellular calcium overload, activation of Ca^2+^-dependent degradation enzymes, and apoptosis. Excess intracellular[Ca^2+^]i can lead to its overload in mitochondria, which can cause mitochondrial dysfunction, increase ROS, and eventually lead to oxidative stress [19].

A number of studies on this subject have been conducted worldwide. Considering the effects of kinetin on mouse hippocampal neurons, Wei et al. found that kinetin can reverse the neurooxidative toxicity induced by glutamate, prevent cell death, and reduce Ca^2+^ influx and ROS accumulation by promoting Nrf2 nuclear translocation and HO-1 expression [20]. Recent studies have shown that pyrethrin-3 O-glucosides can inhibit the apoptosis of neuroblastoma cells induced by *β*-amyloid protein 1-40 [21]. [Ca^2+^]i was found to decrease significantly and the expression of Nrf2 and HO-1 mRNA was increased after treatment with C3G. This suggests that the neuroprotective effect may be partly due to the inhibition of apoptosis by the reduction of the calcium influx and the maintenance of [Ca^2+^]i homeostasis. Glutamate elicits Ca^2+^ signals and workloads that regulate neuronal fate both in physiological and pathological circumstances. Both mechanisms induced by excitotoxic glutamate rely on ROS production and on the change in [Ca^2+^]i [22].

### 2.2. Regulation of Mitochondrial Oxidative Stress

Incubation of neuronal cells with fingolimod (FP) was found to alleviate Vitk3-induced toxicity by decreasing mitochondrial ROS production [23]. Furthermore, this increased the expression and activity of protective factors (expression of Nrf2, HO-1, and Trx2, and activity of GST and NQO1), suggesting that the Nrf2-HO-1 signaling axis mechanism may be related to the inhibition of ROS production in mitochondria.

Piantadosi proved that the Nrf2/HO-1 axis can increase CO production, upregulate SOD2 expression, inactivate glycogen synthase kinase-3 (GSK-3), and induce the expression of mitochondrial biosynthesis and antioxidant genes at the same time, thereby resulting in resistance to doxorubicin-mediated mitochondrial injury and cardiomyocyte death. These results suggest that Nrf2/HO-1 can modulate mitochondrial structure and function in order to resist oxidative stress injury [24].

DOX-treated H9C2 cells showed significantly increased mitochondrial superoxide generation and diminished the cellular antioxidant status. Neferine pretreatment activated IGF-1R signaling, improved the cellular antioxidant pool, increased the expression of downstream targets of IGF-1R (such as PI3K/Akt/mTOR), and significantly inhibited mitochondrial superoxide generation and autophagy with the induction of Nrf2 translocation and HO-1 and SOD1 expression [25].

### 2.3. Regulation of Autophagy

Autophagy is a natural protection mechanism for regulating the internal environment of the body. When stimulated by external injury, proper induction of autophagy can reduce injury to the body [26]. Lin et al. discovered a crosstalk between AMPK-activated autophagy and the Nrf2 signaling axis in lycopene- (LYC-) mediated nephroprotection against atrazine- (ATR-) induced toxicity in mouse kidneys [27]. Yao found that the activation of the Nrf2/HO-1 signaling axis can prevent vascular calcification induced by hyperphosphatemia by inducing autophagy in renal vascular smooth muscle cells [28].

Interestingly, when the overactivation of autophagy harms the structure and function of organs, the Nrf2/HO-1 axis can also inhibit this response for protection. For example, Chung et al. induced the expression of HO-1 and other antioxidant response elements in a rat model of unilateral ureteral obstruction (UUO), proving that these elements protect the functional integrity of mitochondria, decrease ROS production, and downregulate the autophagy-associated protein Beclin-1. Thus, the Nrf2/HO-1 axis helps in protection against autophagy while the Nrf2 knockout increases autophagic cell death [29].

### 2.4. Regulation of Ferroptosis

Ferroptosis is a new type of iron-dependent nonapoptotic cell death model that was recently discovered and found to be closely related to ROS release [30]. Li et al. found that ferroptosis plays a critical role in radiation-induced lung fibrosis (RILF); the ferroptosis inhibitor liproxstatin-1 alleviated RILF through the downregulation of TGF-1 by activating the Nrf2/HO-1 axis [31]. Sun et al. found that the Nrf2 status is a key factor that determines the therapeutic response to ferroptosis-targeted therapies in hepatocellular carcinoma (HCC) cells [32]. Based on the above research, we may conclude that the Nrf2/HO-1 signaling axis has a suppressive effect on ferroptosis. However, as Nrf2 has both positive and negative effects on tumors, its effects on ferroptosis need to be further explored.

### 2.5. Regulation of Pyroptosis

Pyroptosis is a kind of programmed cell necrosis mainly mediated by caspase-1, characterized by changes in cell osmotic pressure, cell membrane rupture, and release of inflammatory substances, resulting in cell death. Inflammasomes are the central regulators of inflammation [33]. Upon detection of various stress factors, the assembly of the inflammasome protein complex results in the activation and secretion of proinflammatory cytokines. In addition, inflammasome activation causes pyroptosis, a lytic form of cell death, which supports inflammation. The NLRP3 inflammasome could exert an inflammatory effect by inducing the secretion of proinflammatory cytokines (i.e., IL-1*β*, IL-18), or could cause pyroptosis in a caspase-1-dependent manner [28]. There is growing evidence of a crosstalk between the Nrf2 and inflammasome axes at different levels. For example, Nrf2-activating compounds inhibit inflammasomes and consequently inflammation. Ferulic acid upregulated both PPAR*γ* and Nrf2 signaling and prevented ROS overproduction, while suppressing the NF-*κ*B/NLRP3 inflammasome axis and apoptosis in the kidney of MTX-induced rats [34].

A dihydromyricetin (DHM) pretreatment inhibited pyroptosis induced by palmitic acid (PA), reduced ROS and mtROS levels, and activated the Nrf2 signaling axis. In addition, the siRNA knockout of Nrf2 could eliminate the inhibitory effect of DHM on ROS production and its subsequent PA-induced focal eye disease. Thus, it is suggested that the Nrf2 signaling axis plays at least a partial role in the reduction of PA-induced pyrolysis of vascular endothelial cells mediated by DHM [29].

### 2.6. Regulation of Programmed Cell Necrosis

Programmed cell necrosis refers to the suicide protection measures initiated by gene regulation when cells are stimulated by internal and external environmental factors, including the activation of some molecular mechanisms and gene programming to remove nonessential cells or cells that are about to be specialized in the body. Chronic exposure to sodium arsenite (NaAsO_2_) abruptly increased lactate dehydrogenase (LDH) release in bronchoalveolar lavage fluid, generated ROS, impaired the antioxidant defense, and distorted the alveolar architecture. These effects could be suppressed by the anti-inflammatory activity of mangiferin in lung tissues [35]. Mangiferin significantly restored the antioxidant balance and inhibited apoptosis in lungs by upregulating the Nrf2/HO-1 axis.

Silencing of Nrf2 by siRNA significantly blocked the cytoprotective effects of sulforaphane (SFP) against dexamethasone- (Dex-) induced apoptosis, suggesting an important role of the Nrf2 signaling axis in cell apoptosis induced by Dex [36]. SFP can significantly inhibit caspase-dependent apoptosis and mitochondrial-mediated apoptosis, both induced by Dex [36]. The excessive production of ROS induced by treatment with Dex treatment inhibited the expression of Nrf2 and downstream effect factors HO-1 and NQO1, which could be effectively reversed by cotreatment with SFP. In addition, siRNA silencing of Nrf2 significantly blocked the protective effect of SFP on Dex-induced apoptosis, suggesting that there is an important correlation between the Nrf2 signaling axis and Dex-induced apoptosis.

Treatment with paraquat (PQ) strongly inhibited the expression of Nrf2 and its downstream effectors, HO-1 and NQO1 [37], while cotreatment with cycloartenyl ferulate (CF) effectively reversed this effect of PQ. However, Nrf2 silencing by siRNA significantly blocked the cytoprotective effects of CF against PQ-induced apoptosis, suggesting an important role of the Nrf2 signaling axis in cell apoptosis induced by PQ.

### 2.7. Research Prospects on Alkaliptosis

Alkaliptosis is a newly found, unique pH-dependent form of regulated cell death (RCD). Tang et al. first introduced the definition of alkaliptosis when they found that the N-acetyl cysteine- (NAC-) mediated acidic pH environment protects against drug-induced cell death, whereas alkalinization of cell culture medium by sodium hydroxide is more likely to induce cell death through NF-*κ*B [38]. This mechanism is typically different from other forms of nonapoptotic regulated cell death including ferroptosis, pyroptosis, and necrosis.

Interestingly, NF-*κ*B expression can both suppress alkaliptosis and induce the Nrf2/HO-1 signaling axis to transactivate proinflammatory and antiapoptotic genes [39]; however, Nrf2 knockdown has not been found to affect drug-induced cell death so far [40]. Thus, there is still much to explore regarding the upstream and downstream regulators in the Nrf2/HO-1 axis that mediate protection against alkaliptosis.

### 2.8. Research Prospects on Clockophagy

The circadian rhythm is a kind of endogenous oscillation mechanism that controls various cellular processes including iron metabolism, oxidative stress, and cell death [34]. Yang et al. demonstrated a novel selective autophagy pathway in tumor cells that promotes the autophagic degradation of ARNTL, the core protein of clockophagy, and promotes ferroptosis of tumor cells [41]. Selective degradation of aryl hydrocarbon receptor nuclear translocator-like protein 1 (ARNTL) by autophagy is the key to ferroptosis. Additionally, Sun et al. found that the activation of the p62-Keap1-Nrf2-HO-1 signaling axis contributes to protection against ferroptosis in carcinoma cells [32]. According to this theory, Nrf2 activation may protect against ferroptosis through ARNTL protein degradation, but there is yet no evidence regarding how the Nrf2/HO-1 axis is involved in the regulation of clockophagy.

## 3. Up-To-Date Clinical Research in Pediatric Respiratory Diseases

The Nrf2/HO-1 axis has different regulatory modes in different respiratory diseases (Table 1).

### 3.1. Pneumonia

Pneumonia is a common lower respiratory tract infection in pediatrics. Reducing the damage caused by pulmonary inflammation and stabilizing lung barrier function are important research directions in the disease treatment.

Konrad et al. used a mouse pneumonia model and found that HO-1 significantly reduces the migration of polymorphonuclear leukocyte disease in the lungs through adenosine receptors 2A and 2B [42]. Wu et al. pointed out that the Nrf2/HO-1 axis inhibits ROS production induced by lipopolysaccharides (LPS) and decreases the expression of inflammatory mediators IL-1*β* and TNF-*α*, so as to alleviate lung injury [43]. Moreover, it reduces the migration of CXCL1-related polymorphonuclear white blood cells to the alveolar septum, reduces microvascular endothelial permeability, and stabilizes lung barrier function [44].

As a result, the Nrf2/HO-1 signaling axis mainly plays an anti-inflammatory role in pneumonia, and the induction of HO-1 expression may be a possible therapy in its clinical treatment.

### 3.2. Asthma

With the acceleration of modernization and urbanization, the incidence of allergic diseases in children and adolescents is also rising rapidly. The overall prevalence of asthma in China has reached 4.2%, and it is estimated that the number of pediatric asthma patients in China will reach 400 million by 2025 [45]. Asthma is a severe condition against which drugs are no longer sufficiently effective, requiring a shift from the previous traditional therapies.

Ano et al. discovered that a lack of Nrf2 prolonged the inflammatory response and airway hyperreactivity after chlorine exposure in mice [46], indicating that Nrf2 prevents airway dysfunction by regulating antioxidant genes such as *TBXAS1*, *GCLC*, *AKR1D1*, and *POR* [47]. Zhang et al. observed that Nrf2 activation could improve O_3_-induced asthma by suppressing ROS and Th17 cells [48]. McGovern et al. recently revealed that some organic dust causing Nrf2 activation can be taken up by bronchial epithelial cells [49].

Consequently, Nrf2 induction can not only downregulate serum levels of IgE along with IL-4, IL-5, IFN-*γ*, and IL-13 but also reduce inflammatory cell infiltration and airway epithelium thickening in the perivascular area [50]. We are pleased to see breakthroughs in asthma treatment gradually being made in exploration of the Nrf2/HO-1 axis. However, Nrf2 or HO-1-mediated drugs are still distant from being introduced in the clinical treatment of asthma.

### 3.3. Lung Neoplasm

Lung neoplasm patients often have a poor prognosis with a dismal 5-year survival rate; existing drugs are not sufficient to effectively control and improve the status of lung cancer patients. Therefore, the development of new drugs for prevention and improvement of the clinical treatment of this disease could be inspired by the Nrf2/HO-1 findings [51].

Yu et al. demonstrated that the inhibition of the Nrf2/HO-1 axis makes non-small-cell lung cancer sensitive to epigallocatechin-3-gallate (EGCG) therapy [52], as HO-1 promotes resistance to EGCG-induced apoptosis. However, the effect of Nrf2 is controversial as it has a dual function depending on different lung cancer stages. On one hand, upregulated Nrf2 expression retards malignant lung cancer cell transformation [53]. On the other hand, Nrf2 is involved in various carcinogenic signaling axes and is related to other transcription factors and structural proteins in cancer pathogenesis [54].

Recent studies also summarize preclinical evidence suggesting that the induction of alkaliptosis and ferroptosis may be effective in human cancer treatment [40]. Understanding the acid-base balance patterns of cancer cells and the anticancer activity of ferroptosis activators may bring new insights into the phenotypes and provide directions when considering Nrf2/HO-1-targeted lung cancer therapy [41].

### 3.4. Acute Respiratory Distress Syndrome (ARDS)

ARDS is a clinical syndrome mainly characterized by progressive dyspnea and pulmonary hypertension, permeability pulmonary edema, and pulmonary microthrombosis [55].

The levels of TNF-*α*, IL-1*β*, and IL-6 were significantly increased in the serum and bronchoalveolar lavage fluid of ARDS patients, suggesting that these cytokines may play an important role in the development of the disease. The Nrf2/HO-1 signaling axis can antagonize TNF-*α*-mediated injury of capillary endothelial cells, thereby improving the barrier function of capillary endothelial cells, reducing capillary permeability, and stimulating endothelial cells to release a large number of anti-inflammatory cytokines [56].

Yan et al. recently found that antioxidants, to some extent, protect the cell from oxidative damage induced by H_2_O_2_ by regulating the apoptosis mediated by Nrf2 [57]. Further, Pereira et al. discussed that HO-1 may be a probable biomarker for the severity of ARDS caused by malaria, as it controls inflammation and pulmonary vascular permeability [58, 59].

### 3.5. Idiopathic Pulmonary Fibrosis (IPF)

IPF is a chronic, progressive, fibrotic interstitial pulmonary disease that can eventually lead to pulmonary interstitial fibrosis and irreversible respiratory failure or even death. Sadly, there is no specific treatment so far that improves the disease's prognosis and survival rate. It is universally acknowledged that oxidative stress is a vital regulating factor of idiopathic pulmonary fibrosis, and Chitra et al. demonstrated that the dysregulation of Nrf2/HO-1, the main regulator of oxidative stress, largely leads to pulmonary fibrosis [60]. This suggests that the inhibition of oxidant stress through the induction of HO-1 expression may be a future research direction.

In particular, pulmonary fibrosis induced by organophosphorus pesticides such as PQ has been a typical threat in underdeveloped regions. Tai et al. found that rapamycin alleviates PQ-induced pulmonary fibrosis by activating the Nrf2/HO-1 axis both *in vivo* and *in vitro* [61]. As a result, research on the Nrf2/HO-1 axis may possibly improve public health conditions in underdeveloped areas in the long run.

### 3.6. Chronic Obstructive Pulmonary Diseases (COPD)

COPD is mainly related to the shifting of the Th1/Th2 ratio to the Th1 end. Studies have reported acute COPD patients with elevated Th2 cells [62]. This is because bacteria, allergens, and other external factors stimulate the airway to produce airway inflammation. Infection and allergy can induce the activation and increment of Th2 cells [62]. In our previous studies, we analyzed the effective role that HO-1 plays in regulating the Th1/Th2 ratio and inducing the secretion of anti-inflammatory cytokines [56].

Recently, more studies have been conducted on this subject. Yuichi et al. also found that Nrf2 and HO-1 affect the expression of antioxidant genes and the degree of inflammation after influenza infection in mice exposed to cigarette smoke [63]. These results highlight the anti-inflammatory effects of the Nrf2/HO-1 axis in COPD. According to Liu et al., cigarette smoke can cause significant pulmonary inflammation and apoptosis of alveolar cells [53]. Oxidant burden and apoptosis due to the suppression of Nrf2 expression in the lungs can have severe results, such as COPD. Thus, the development of novel therapeutic strategies for pediatric respiratory diseases is urgently needed. Nrf2 mediates the protective effects of both Wnt3a/*β*-catenin and AMPK on lung inflammatory responses during the development of COPD/emphysema [64].

## 4. Nrf2 as a Potential Redox Biomarker

It has long been believed that the putative perturbation of redox signals is a key factor in the expression of oxidative stress [65]. Scapagnini et al. have found that plant-derived compounds are Nrf2 inducers that can upregulate the expression of antioxidant and detoxification genes [66]. Veskoukis et al. recently proposed that Nrf2 can be used a translational biomarker to evaluate the internal and external antioxidant effects of plant polyphenols [67]. This indicates that Nrf2 may be considered as a biomarker to evaluate oxidative stress. In light of these results, these oxidative stress markers should be functionally grouped in order to be applied to conventional toxicological measurements [65]. Hopefully, if Nrf2 indeed proves to be an oxidative stress biomarker, it could be used as a clinical diagnostic indicator of pediatric respiratory diseases in the future.

As previously described, the Nrf2/HO-1 signaling axis inhibits oxidant stress in pneumonia, asthma, idiopathic pulmonary fibrosis, and chronic obstructive pulmonary diseases. These results indicate that Nrf2 can not only alleviate diseases through its upregulated expression but also serve as a comparative indicator of disease diagnosis, treatment status, and prognosis. Moreover, pediatric respiratory diseases such as pneumonia and asthma are related to oxidative stress, and we may improve the quality of life of the children by implementing antioxidative stress methods.

## 5. Conclusions

The Nrf2/HO-1 signaling axis has a complex regulatory mechanism in oxidative stress diseases, exerting anti-inflammatory and antioxidant effects. It is involved in the reduction of mitochondrial damage and in the regulation of Ca^2+^ intracellular flow and cell death, which are indispensable signaling axes for protection against oxidative stress. Nrf2 plays a complex and varied role in human diseases and can prevent the occurrence of chronic diseases and cancer, as well as contribute to the survival of cancer cells and make tumor cells resistant to radiotherapy and chemotherapy [68, 69]. The dual effects of both Nrf2 and HO-1 suggest that the regulatory mechanisms of this signaling axis are still not fully explored. In-depth exploration of the pathogenesis of Nrf2/HO-1 in pediatric respiratory diseases can thus provide a molecular basis for research on targeted drug therapy and help realize the potential value of this axis in clinical drug research.

## Figures and Tables

**Table 1:** Mechanisms of the Nrf2/HO-1 signaling axis in pediatric respiratory diseases.

Pediatric respiratory disease	Effects	Reference
Pneumonia	Reduces the migration of polymorphonuclear leukocytes in the lung through adenosine receptors 2A and 2B	[42]
	Inhibits ROS production and decreases the expression of inflammatory mediators IL-1*β* and TNF-*α*	[43]
	Reduces the migration of CXCL1-related polymorphonuclear white blood cells to the alveolar septum	[44]

Asthma	Regulates antioxidant genes	[46]
	Suppresses ROS and TH17 cells	[48]
	Downregulates serum levels of IgE, IL-4, IL-5, IFN-*γ*, and IL-13	[50]

Lung neoplasm	Promotes resistance to apoptosis induction	[52]
	Retards malignant lung cancer cell transformation	[53]
	Induces alkaliptosis and ferroptosis	[40, 41]

Acute respiratory distress syndrome	Releases a large number of anti-inflammatory cytokines	[56]
	Improves the barrier function of capillary endothelial cells and reduces capillary permeability	[57]
	Is a probable biomarker of ARDS severity	[59]

Idiopathic pulmonary fibrosis	Inhibits the degree of oxidant stress	[60]
	Decreases the upregulated effects of cell death and apoptosis, expression of fibrosis-related factors, and transformation of fibroblasts to myofibroblasts	[61]

Chronic obstructive pulmonary diseases	Regulates the Th1/Th2 ratio and induces secretion of anti-inflammatory cytokines	[56]
	Affects the expression of antioxidant genes	[63]
	Reduces the burden of oxidation in the lung and reduces apoptosis	[53]
